# Alterations of *Bacteroides* sp*.*, *Neisseria* sp*.*, *Actinomyces* sp*.*, and *Streptococcus* sp*.* populations in the oropharyngeal microbiome are associated with liver cirrhosis and pneumonia

**DOI:** 10.1186/s12879-015-0977-x

**Published:** 2015-06-23

**Authors:** Haifeng Lu, Guirong Qian, Zhigang Ren, Chunxia Zhang, Hua Zhang, Wei Xu, Ping Ye, Yunmei Yang, Lanjuan Li

**Affiliations:** State Key Laboratory for Diagnosis and Treatment of Infectious Diseases, Collaborative Innovation Center for Diagnosis and Treatment of Infectious Diseases, The First Affiliated Hospital, College of Medicine, Zhejiang University, 79 Qingchun Road, Hangzhou, 310003 People’s Republic of China; Tonglu First People’s Hospital, 338 Xuesheng Road, Tonglu, Hangzhou, 311500 People’s Republic of China; Department of Geriatrics, The First Affiliated Hospital, School of Medicine, Zhejiang University, 79 Qingchun Road, Hangzhou, 310003 People’s Republic of China

**Keywords:** Whole genome amplification (WGA), Oropharyngeal microbiome, Pneumonia, Cirrhosis, Denaturing gradient gel electrophoresis (DGGE)

## Abstract

**Background:**

The microbiomes of humans are associated with liver and lung inflammation. We identified and verified alterations of the oropharyngeal microbiome and assessed their association with cirrhosis and pneumonia.

**Methods:**

Study components were as follows: (1) determination of the temporal stability of the oropharyngeal microbiome; (2) identification of oropharyngeal microbial variation in 90 subjects; (3) quantitative identification of disease-associated bacteria. DNAs enriched in bacterial sequences were produced from low-biomass oropharyngeal swabs using whole genome amplification and were analyzed using denaturing gradient gel electrophoresis analysis.

**Results:**

Whole genome amplification combined with denaturing gradient gel electrophoresis analysis monitored successfully oropharyngeal microbial variations and showed that the composition of each subject’s oropharyngeal microbiome remained relatively stable during the follow-up. The microbial composition of cirrhotic patients with pneumonia differed from those of others and clustered together in subgroup analysis. Further, species richness and the value of Shannon’s diversity and evenness index increased significantly in patients with cirrhosis and pneumonia versus others (*p* < 0.001, versus healthy controls; *p* < 0.01, versus cirrhotic patients without pneumonia). Moreover, we identified variants of *Bacteroides*, *Eubacterium*, *Lachnospiraceae*, *Neisseria*, *Actinomyces,* and *Streptococcus* through phylogenetic analysis. Quantitative polymerase chain reaction assays revealed that the populations of *Bacteroides*, *Neisseria*, and *Actinomycetes* increased, while that of *Streptococcus* decreased in cirrhotic patients with pneumonia versus others (*p* < 0.001, versus Healthy controls; *p* < 0.01, versus cirrhotic patients without pneumonia).

**Conclusions:**

Alterations of *Bacteroides*, *Neisseria*, *Actinomyces,* and *Streptococcus* populations in the oropharyngeal microbiome were associated with liver cirrhosis and pneumonia.

**Electronic supplementary material:**

The online version of this article (doi:10.1186/s12879-015-0977-x) contains supplementary material, which is available to authorized users.

## Background

Liver disease and associated complications represent a major healthcare burden in China. Pneumonia is a far more common complication in patients with decompensated cirrhosis. Increasing awareness of the role of the microbiome of humans in the progression of liver [1] and lung inflammation [2, 3] raises the importance of assessing the composition of the respiratory microbiome as well as the nature of disease-induced changes caused by these microbiomes during disease progression. Current treatment strategies for pneumonia are based on the routine identification of cultured bacteria, including the isolation of clinically significant bacterial species from sputum. However, the upper and lower respiratory tracts harbor a vast range of commensal and potentially pathogenic bacteria that forms an indispensable part of the human microbiome [4]. These organisms live in a complex, yet balanced relationship, and therefore manipulation of one may affect the other members of the community. Although the presence of pathogens is a prerequisite for infections and alterations of the community, which may lead to overgrowth and invasion, it is a key factor leading to infection [5].

Studying the microbial variations of patients with pneumonia is challenging, because sampling the lung microbiome requires an invasive surgical procedure that may harm subjects. The oropharynx serves the respiratory and digestive systems and is colonized by bacterial pathogens that affect healthy or immunocompromised individuals [6]. The importance of the microbial composition of the oral and respiratory tracts is increasingly considered as a source of biomarkers to facilitate noninvasive detection of disease [7, 8]. A better understanding of the significance of variations of the oropharyngeal mucosal microbiome in preclinical conditions and disease may provide insights into selective oropharyngeal decontamination that is used to prevent susceptible patients from contracting pneumonia [9]. Moreover, samples are acquired with minimal disturbance of the microbiome, and the technique exposes patients to minimal risk.

Denaturing gradient gel electrophoresis (DGGE) is a DNA fingerprinting technique used to accurately assess the members of a microbial community by generating patterns or profiles of genetic diversity [10]. DGGE facilitates rapid analyses and comparisons of microbial communities. Moreover, polymerase chain reaction (PCR) amplicons are isolated from the DGGE profile, which can be further amplified and sequenced [10, 11]. Therefore, it is a preferred technique for studies of the composition, structure, and stability of complex microbial communities. However, the low biomass obtained from oropharyngeal swabs impedes the application of the DGGE technique for characterizing microbial populations of the oropharyngeal mucosa. Whole genome amplification (WGA) using phi29 DNA polymerase overcomes insufficient sample sizes [12]. For example, numerous studies of microbial ecology show that WGA using phi29 DNA polymerase enriches environmental template DNAs with the highest amplification efficiency [13, 14], and the amplified DNA can be used to characterize the microbial community structure in low-biomass environments [15–17]. Here, we applied phi29 DNA polymerase to generate sufficient quantities of DNA from oropharyngeal mucosal swabs to analyze and monitor the microbial community associated with liver cirrhosis and pneumonia, which was accompanied by further DGGE analysis of the V3 region of the 16S rDNA. We aimed to conduct a preliminary assessment of the variations of the predominant oropharyngeal mucosal among cirrhotic patients with or without pneumonia and healthy controls as well as the microbial alterations during the follow-up. Our ultimate goal was extend our knowledge of the contributions of the respiratory microbiome in the human lung in health and disease.

## Methods

### Study design, patients, and samples

From September 2011 to August 2012, the study recruited 90 subjects, including 30 with hepatitis B virus (HBV)-decompensated cirrhosis with confirmed pneumonia (Group CI), 30 with HBV-decompensated cirrhosis without respiratory infection (Group CC), and 30 healthy adult controls matched for age and sex to the cirrhotic group (Group HC). The clinical characteristics collected from medical records of the subjects are shown in Table 1. A subset of subjects was randomly chosen to provide swab specimens every 5 days within the 3-week follow-up period after a positive swab confirmation. The follow-up study included 12 patients with HBV-decompensated cirrhosis with pneumonia, 10 without respiratory infection, and 10 healthy controls. None of the controls had a detectable infection during the follow-up. The patients fulfilled the criteria as follows: (i) liver cirrhosis detected using ultrasound with or without pneumonia, which was confirmed using computerized tomography of the chest; (ii) physician’s diagnosis of ascites, gastrointestinal bleeding, and malnutrition; (iii) not treated with antibiotics, lactulose, prokinetic drugs, or proton pump inhibitors 3 weeks before hospitalization; (iv) absence of other organ dysfunction, except the liver, lung, and previous related surgery. Exclusion criteria included other infections such as peritonitis and bacteremia confirmed using ascites fluid and routine bacteriological analysis of blood. A physician supervised the collection of the first swab specimens of the unstimulated oropharynx within 2 h after the patients’ hospitalization, and antibiotics were not administered. Written informed consent and questionnaires (Additional file 1: Table S1) were obtained from all subjects who voluntarily underwent sample collection using oropharyngeal mucosal swabs. The Ethics Committee of the First Affiliated Hospital, School of Medicine, Zhejiang University approved this study.

**Table 1:** Subject characteristics

Characteristics	Group HC (*n* = 30)	Group CC (*n* = 30)	Group CI (*n* = 30)
Age	62.80 ± 3.19	63.47 ± 2.29	65.23 ± 3.42
Sex: Male/female	23/7	21/9	22/8
Hospital days	-^a^	10.5	25.5
Primary blood parameters at first follow-up:			
WBC (10^9^/L)	8.48 ± 1.13	7.72 ± 5.33	14.60 ± 18.47
CRP (mg/L)	<10	21.97 ± 7.54	136.02 ± 79.39
ALB (g/L)	50.19 ± 2.84	29.97 ± 7.54	32.88 ± 3.31
ALT (U/L)	18.77 ± 7.65	140.53 ± 53.04	117.55 ± 30.27
AST (U/L)	17.90 ± 5.26)	173.07 ± 62.10)	109.29 ± 38.47
TBIL (mol/L)	9.63 ± 1.65	49.03 ± 16.63	51.37 ± 23.44
Infection with			
*Candida*	-	-	5
*Streptococcus pneumoniae*	-	-	14
*Mycoplasma pneumoniae*	-	-	3
*Pseudomonas aeruginosa*	-	-	3
*Staphylococcus*	-	-	5

Mean ± SD

*WBC* white blood cell, *CRP* C-reactive protein, *ALB* albumin, *ALT* alanine aminotransferase, *AST* aspartate aminotransferase, *TBIL* total bilirubin

^a^Not applicable

### Sampling and DNA extraction

Microbial samples were obtained from the posterior wall of the oropharynx using sterile Copan swabs (Copan Diagnostics Inc., California, USA). The swab sample was immersed in phosphate-buffered saline, transferred to the laboratory, shaken, centrifuged immediately, and the supernatant was discarded. The pellets were stored at −80 °C within an hour. Microbial DNA was extracted using a Qiagen Mini Kit (Qiagen, Hilden, Germany) following the manufacturer’s instructions. The DNA was quantified using a Qubit 2.0 Fluorometer (Invitrogen, Carlsbad, CA, USA), and all oropharyngeal microbial DNAs were diluted to 3 ng/μL for WGA.

### Whole genome amplification (WGA)

Bacterial genomic DNA was subjected to multiple displacement amplification using the GenomiPhi V2 DNA Amplification Kit [12] (GE Healthcare, Amersham Place, Buckinghamshire, UK) according to the manufacturer’s protocol. After WGA, the amplicons were quantified using a Qubit 2.0 Fluorometer. Each sample was amplified in triplicate, and the three reaction products per sample were diluted 1:10 and stored at −30 °C.

### PCR amplification of the 16S rDNA V3 region

The V3 variable region of 16S rDNA was amplified using a hot-start touchdown protocol with primers specific for the conserved regions of the 16S rRNA gene [18]. The reaction mixture contained 400 ng of genomic DNA, 25 pmol of each primer, 0.2 μM dNTPs, 1 × Ex Taq buffer, and 2.5 U of Ex Taq polymerase (Takara, Dalian, China), and the final volume was adjusted to 50 μL with sterile deionized water. To minimize hetero duplex formation, five-cycle reconditioning PCR was conducted using 5 μL of amplification mixture in a fresh reaction mixture as previously described [19]. The reamplified products (25 μL) were analyzed using DGGE.

### DGGE profiling

Parallel DGGE profiling was performed as previously described [11, 20]. Electrophoresis was conducted at a constant 70 V at 60 °C for approximately 16 h. DGGE profiles were processed using BioNumerics software version 6.01 (Applied Maths, St-Martens-Latem, Belgium) in a multistep procedure following the manufacturer’s instructions. Parameters for allocating band-classes were set according to Joossens *et al. *[20]. Cluster analysis of DGGE profiles used the unweighted pair-group method with an arithmetic mean (UPGMA) based on the Dice similarity coefficient [21]. Multidimensional scaling (MDS) and principal component analysis (PCA) were conducted according to their respective manuals in BioNumerics software. *Past* software was used to derive the Shannon H scores (diversity index), Shannon’s evenness index, and species richness [22].

The samples were fractionated using 19 gels, and three standard reference lanes were included on the sides and in the middle of each DGGE gel to allow comparisons among gels using BioNumerics software. We excised as many bands as possible from each gel. Gel slices were placed in sterile Eppendorf tubes, washed three times in TE buffer (10 mM Tris-HCl, 1 mM EDTA, pH 8.0), disrupted, and incubated in 50 μL of TE buffer for 30 min at 80 °C. DNA in 5 μL of buffer solution was used as a template for PCR re-amplification with universal bacterial primers F357 + GC clamp and R518 as described above for DGGE. Amplicons were analyzed using DGGE, excised until a single band was obtained, stored at − 20 °C, and verified according to their reference site in DGGE gels by comparing gels using BioNumerics software. Two or three amplicons from the same band-class were selected and purified using a QIA quick PCR purification Kit (Qiagen, Hilden, Germany), ligated to pGEM-T Easy Vector DNA (Promega, Madison, WI, USA), and used to transform competent Escherichia coli DH5. Positive colonies were verified and sequenced (Invitrogen, Shanghai, China). The sequences were deposited into the European Nucleotide Archive (ENA) database (Submission ID: Hx200004778).

Sequence similarities were determined using BLAST to search the GenBank DNA database [14]. Based on the BLAST results, reference sequences of phylogenetic neighboring species (97 % similarity) were included for cluster analysis according to multiple sequence alignments generated using MEGA5 software (Molecular Evolutionary Genetics Analysis version 5) [10].

### Quantitative PCR

Quantification of the bacterial species of interest in each original (non-WGA) sample DNA was performed using qPCR as described previously [11, 20]. The primers (Shanghai Invitrogen Biotechnology Limited Company, Shanghai, China) and annealing temperatures are shown in Additional file 2: Table S2. Quantification of *Lachnospiraceae* and *Bacteroides* was performed using a Taqman assay, and other species-specific assays employed SYBR Green.

The copy number of bacterial species was determined by comparison with serially diluted plasmid DNA standards run on the same plate. The plasmid DNA standards were prepared from known concentrations of plasmid DNA [23]. The protocol to determine the detection limit was performed as described previously [24]. The abundances of bacterial species were expressed as log 10 values per 10 ng original DNA template.

### Statistical analysis

Statistical analysis was conducted according to Joossens *et al.* [10]. Mann–Whitney tests were performed to compare differences in oropharyngeal microbiomes across the groups using SPSS version 17.0 for Windows (SPSS Inc., Chicago, IL, USA).

## Results

### The applicability, feasibility, and validity of the WGA method to enrich microbial DNA

**Fig. 1:**
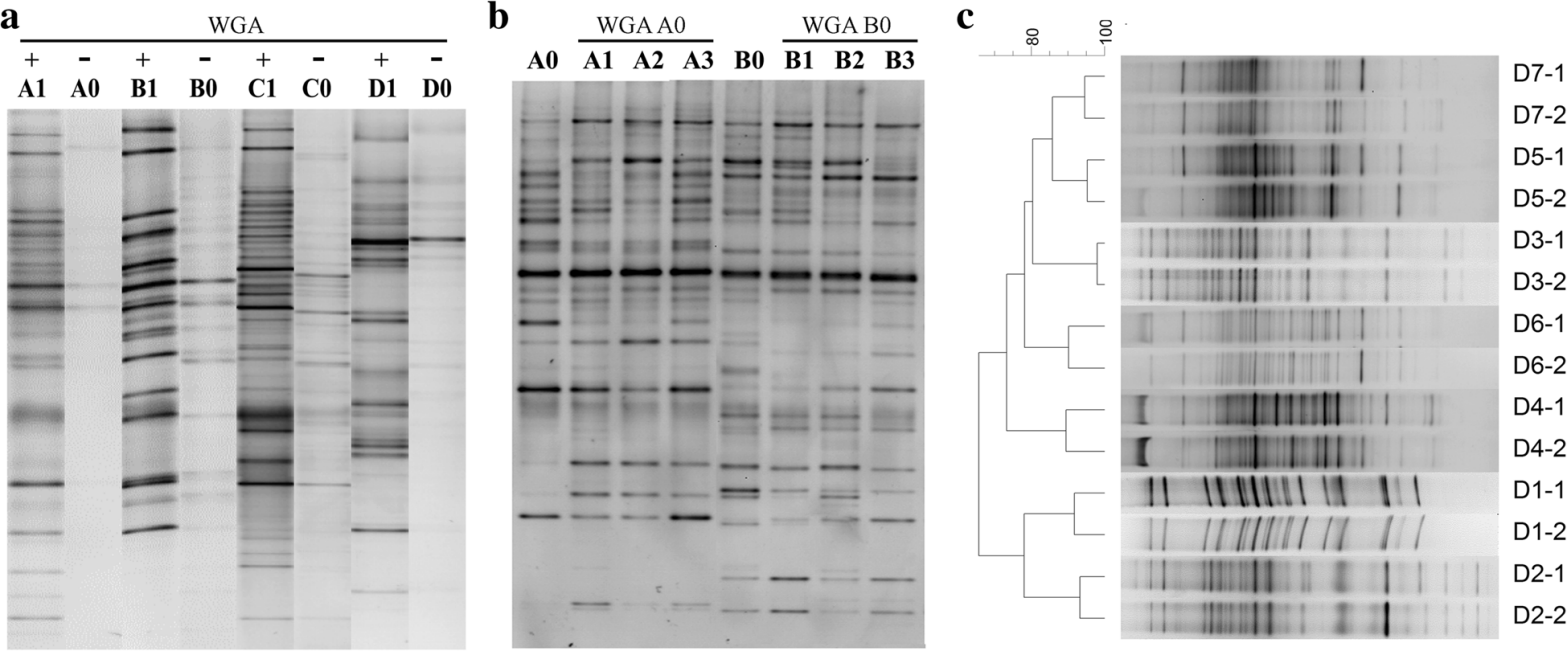
The applicability, feasibility, and validity of the WGA method for enriching microbial DNA. (**a**) DGGE profiles of WGA-amplified oropharyngeal DNAs (lanes A1, B1, C1, and D1) and the original oropharyngeal DNA (lanes A0, B0, C0, and D0); (**b**) the WGA-amplified fecal DNA (lanes A1–3 and B1–3) represented the most predominant microbial bands that were similar to those detected in the original fecal DNA (lanes A0 and B0), and were reproducible; (**c**) Seven oropharyngeal microbial DNA samples were amplified in duplicate using WGA for DGGE analysis. Clustering was performed using Dice’s coefficient and UPGMA with BioNumerics software

We first analyzed the applicability of the WGA method for characterizing oropharyngeal microbial DNA samples. We compared the DGGE profiles between WGA-amplified DNA (Fig. 1a, lanes A1, B1, C1, and D1) and the original DNA (Fig. 1a, lanes A0, B0, C0, and D0) and found that the WGA-positive samples comprised more bands than those of WGA-negative samples, suggesting that the WGA method was capable of enriching oropharyngeal microbial DNA samples for DGGE analysis.

We then determined the reproducibility of the WGA method. Two fecal microbial DNAs were diluted 1:100 (3 ng/μL) and amplified in triplicate using the WGA method. The DGGE profile analysis indicated that the WGA method amplified fecal DNA (Fig. 1b, lanes A1–3 and B1–3), the most predominant bands were similar to those of the original fecal DNA (Fig. 1b, lanes A0 and B0), and the results were reproducible. These results prove that the WGA method effectively enriched low concentrations of microbial DNA suitable for DGGE analysis.

The validity of the WGA method was further characterized using DGGE profiling and cluster analysis. Seven oropharyngeal microbial DNA samples were amplified in duplicate using WGA. Cluster analysis showed that the DGGE profiles were >90 % similar between duplicates (Fig. 1c), suggesting the validity and reproducibility of the WGA method for preparing small quantities of DNA for DGGE analysis.

### Determination of the temporal stability of the oropharyngeal microbiome in a 3- week follow-up study

To investigate the associations between the oropharyngeal microbiome and disease, we first confirmed the temporal stability of oropharyngeal microbiomes of the HC and CC groups during a follow-up period. We chose randomly 32 subjects, including 10 volunteers from the HC group (three times), 10 patients from the CC group (three times, most were discharged from our hospital before the fourth appointment.), and 12 patients from the CI group (four times). The first sample from the patients in the CI group were before antibiotic treatments, the second samples were during antibiotic treatments, and the third and fourth were after antibiotic treatments.

**Fig. 2:**
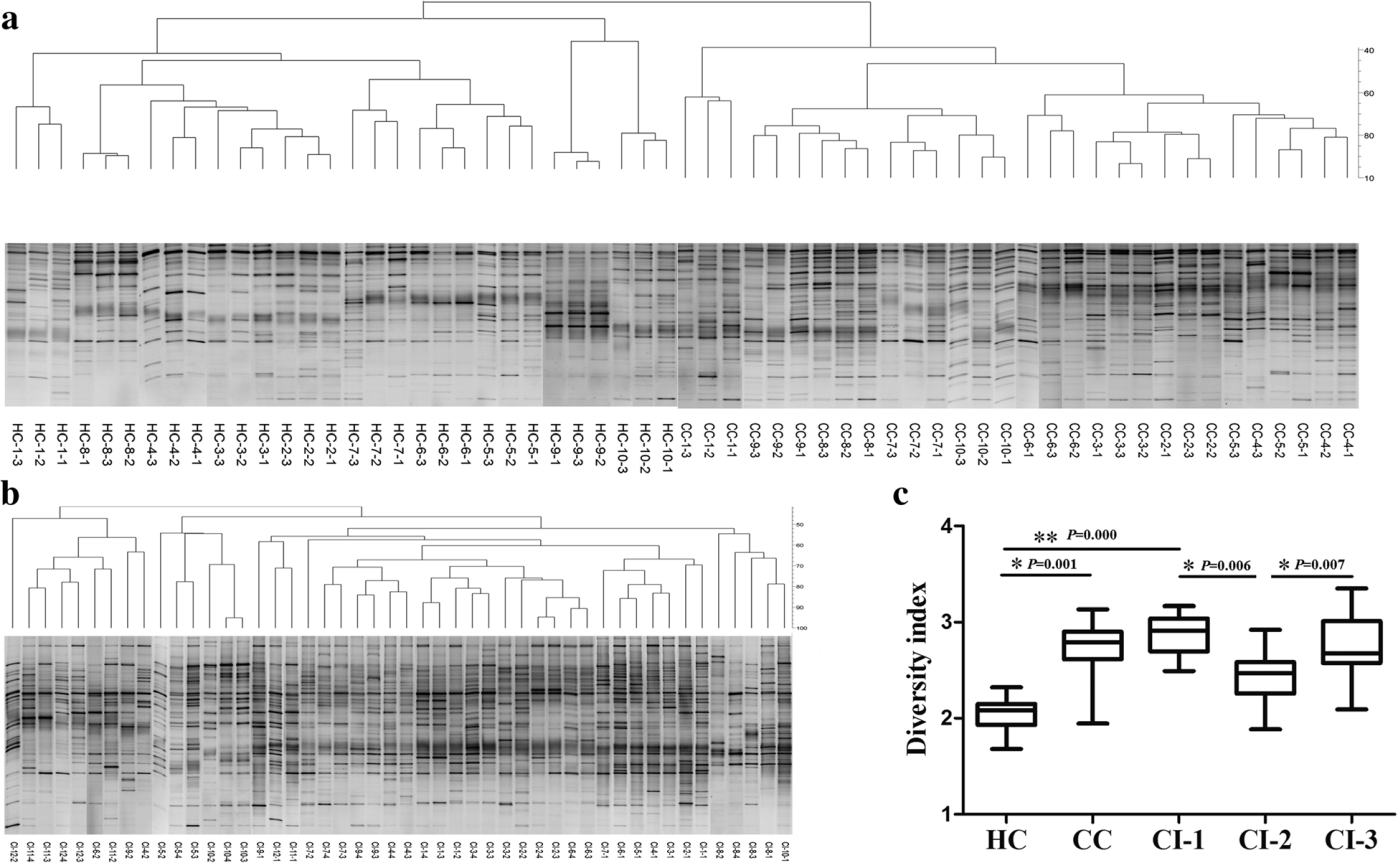
Confirmation of the temporal stability of the oropharyngeal microbiome in a 5-day follow-up study. Cluster analysis of DGGE profiles of oropharyngeal bacteria in groups HC (**a**), CC (a), and CI (b)**.** Cluster analysis was performed using Dice’s coefficient and UPGMA. Lanes were designated by patient number (1, 2, 3, 4), and visit number (1, 2, 3, and 4).CIn-1, before antibiotic treatment; CIn-2, during antibiotic treatment; CIn-3 and -4, after antibiotic treatment. The diversity index of these follow-up samples is shown (c). *p < 0.01; **p < 0.001

The DGGE profiles and cluster analysis demonstrated that three follow-up samples of each individual from the HC or CC groups (*n* = 10) clustered together, respectively (Fig. 2a). These results suggest that the oropharyngeal mucosal microbiome of each control individual exhibited relatively stable patterns during the follow-up (Fig. 2a). In contrast, because of antibiotic treatment, four follow-up samples from patients with pneumonia from the CI group (*n* = 12) did not cluster together (Fig. 2b). However, the DGGE profiles of each patients with pneumonia were similar between their third and fourth visits (Fig. 2b), suggesting that the oropharyngeal microbiome was relatively stable in patients after antibiotics treatments.

Moreover, the diversity of the oropharyngeal microbiome among different groups was calculated using *Past* software. The predominant microbiome of patients with pneumonia from group CI had the highest diversity (CI-1), followed by control patients with liver cirrhosis (group CC) (Fig. 2c). The diversities of groups CI and CC were higher compared with that of the group HC. Notably, oropharyngeal microbial diversity was decreased in group CI-2 during antibiotic treatments versus group CI-1 before antibiotic treatments.

To investigate oropharyngeal microbial variation and identify the key bacteria associated with liver cirrhosis and pneumonia, we analyzed the DGGE profiles of the 90 subjects.

### Cluster analysis of DGGE profiles

**Fig. 3:**
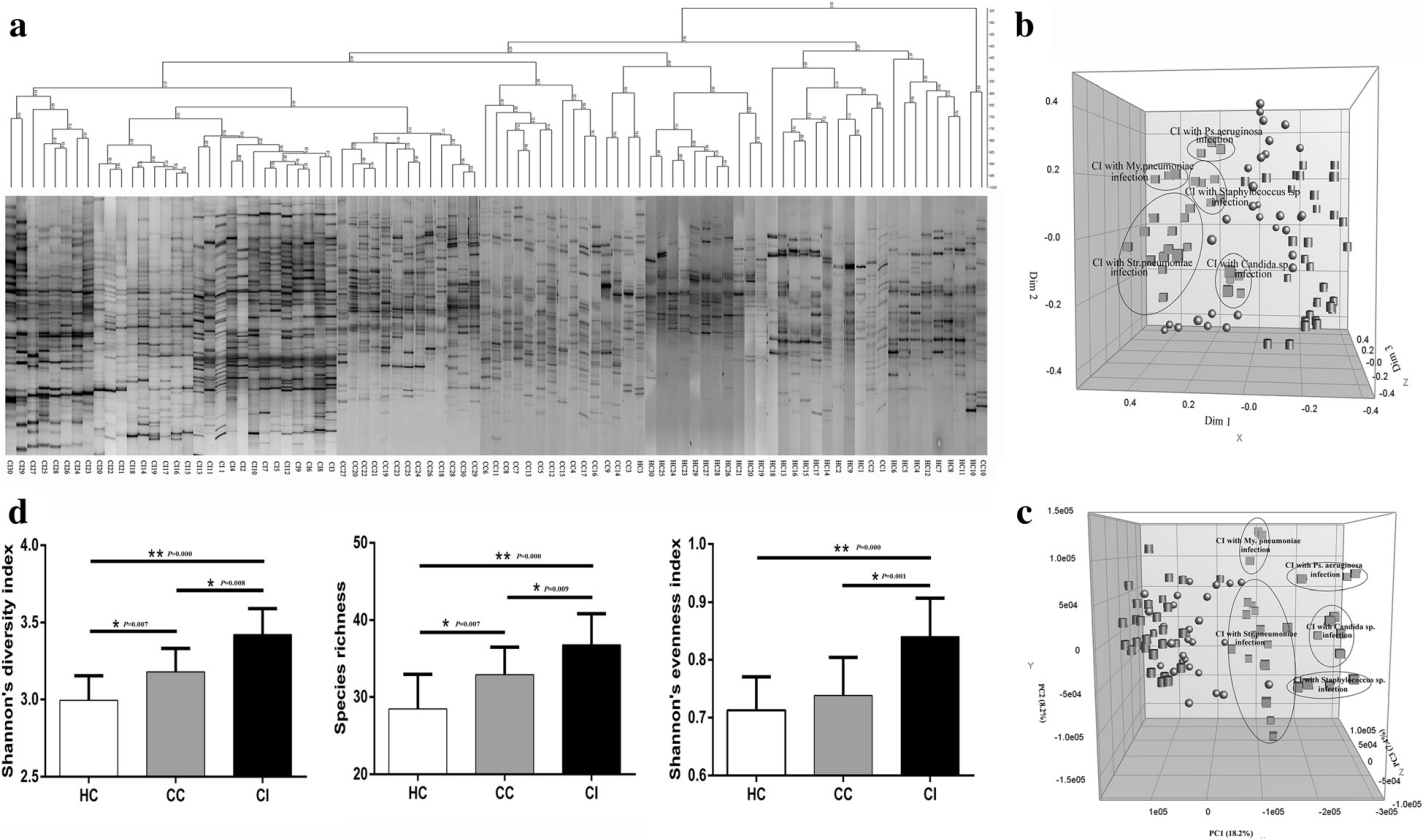
Cluster and diversity analyses of DGGE profiles of the oropharyngeal mucosal microbiomes of 90 PCR-DGGE profiles. (**a**) Cluster analysis of DGGE profiles of the oropharyngeal mucosal microbiome; (**b**) Multidimensional scaling analysis (MDS) based on the predominant oropharyngeal bacterial PCR-DGGE profiles. The plot is an optimized three-dimensional representation of the similarity matrix obtained using BioNumerics software, and the x-, y-, and z-axes represent three different dimensions (Dim 1, Dim 2, and Dim 3, respectively). (**c**) Principal component analysis (PCA) based on bacterial PCR-DGGE profiles. The plots were reoriented to maximize the variation among lanes along the first three principal components (contributions: 18.2, 8.2, and 7.4 %, respectively) obtained using BioNumerics software. Cubes, group CI; spheres, group CC; cylinders, group HC. (**d**) Comparison of the diversity index, species richness, and evenness index of oropharyngeal mucosal microbiomes. *p < 0.01; **p < 0.001

Cluster analysis of DGGE profiles indicates that almost all individuals in each group (except for three samples from the group CC) clustered together, suggesting that the microbial composition of each individual in the same group was similar to the others and that the microbial composition of patients in the group CI differed from those of both control groups. Notably, the DGGE profiles of all patients in group CI clustered together at high UPGMA coefficient values ranging from 57.7 to 94.0 % (average, 82.30 ± 9.85, Fig. 3a). These results were confirmed using MDS analysis (Fig. 3b) and PCA (Fig. 3c). For example, note the overlap of symbols representing the microbiomes of patients infected with the same pathogen in the group CI.

### Analysis of microbial diversity

We used *Past* software to assess the microbial diversity of the oropharyngeal mucosa using Shannon’s diversity index, Species richness, and Shannon’s evenness index. The values of Shannon’s diversity index, Species richness, and Shannon’s evenness index were obviously higher in group CI compared with those in groups CC and HC (*p* < 0.01) (Fig. 3d). Further, Shannon’s diversity index and Species richness were higher in group CC compared with group HC (*p* < 0.01).

#### Phylogenetic analysis of DNAs isolated from DGGE profiles

**Fig. 4:**
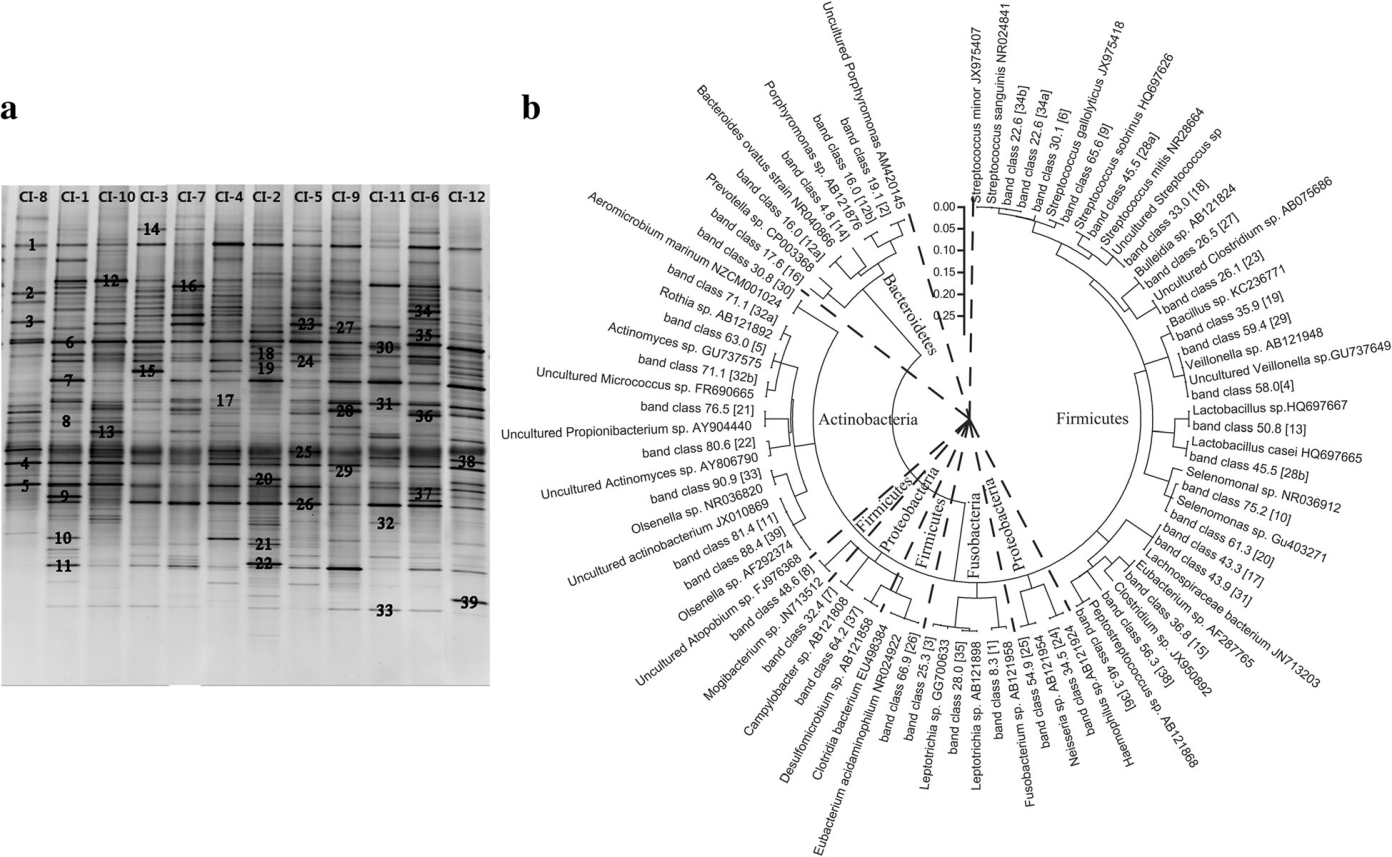
Phylogenetic tree analysis based on bacterial PCR-DGGE profiles. (**a**) Nucleotide sequence identification of the bands from the PCR-DGGE profiles. The number corresponds to the number of band-classes in the phylogenetic tree. (**b**) The phylogenetic tree generated using a neighbor-joining method of the sequences derived from the DGGE profiles. The fragment sequences were designated according to their positions in gels using the band-matching tool with BioNumerics software version 6.01. The plot was prepared using MEGA5 software

In the 90 PCR-DGGE profiles analyzed in this study, 39 band-classes were identified (Fig. 4a). *Firmicutes* (20 band-classes) was the most common phylum, followed by *Actinobacteria* (8 band-classes), *Bacteroidetes* (4 band-classes), *Proteobacteria* (4 band-classes), and *Fusobacteria* (3 band-classes) (Fig. 4b). Seven band-classes were highly prevalent (median intensity in at least one of the groups was >2 %), including band-classes 30.1, 33.0, and 65.6 of *Streptococcus*, band-class 8.3 of *Fusobacterium*, band-class 59.4 of *Veillonella*, as well as band-classes 80.6 and 63.0 of *Actinomyces*, in which there was little variance among band-classes 65.6, 59.4, and 80.6 (Additional file 3: Table S3).

### Identification of bacterial species that account for the variation in the oropharyngeal microbiome

**Fig. 5:**
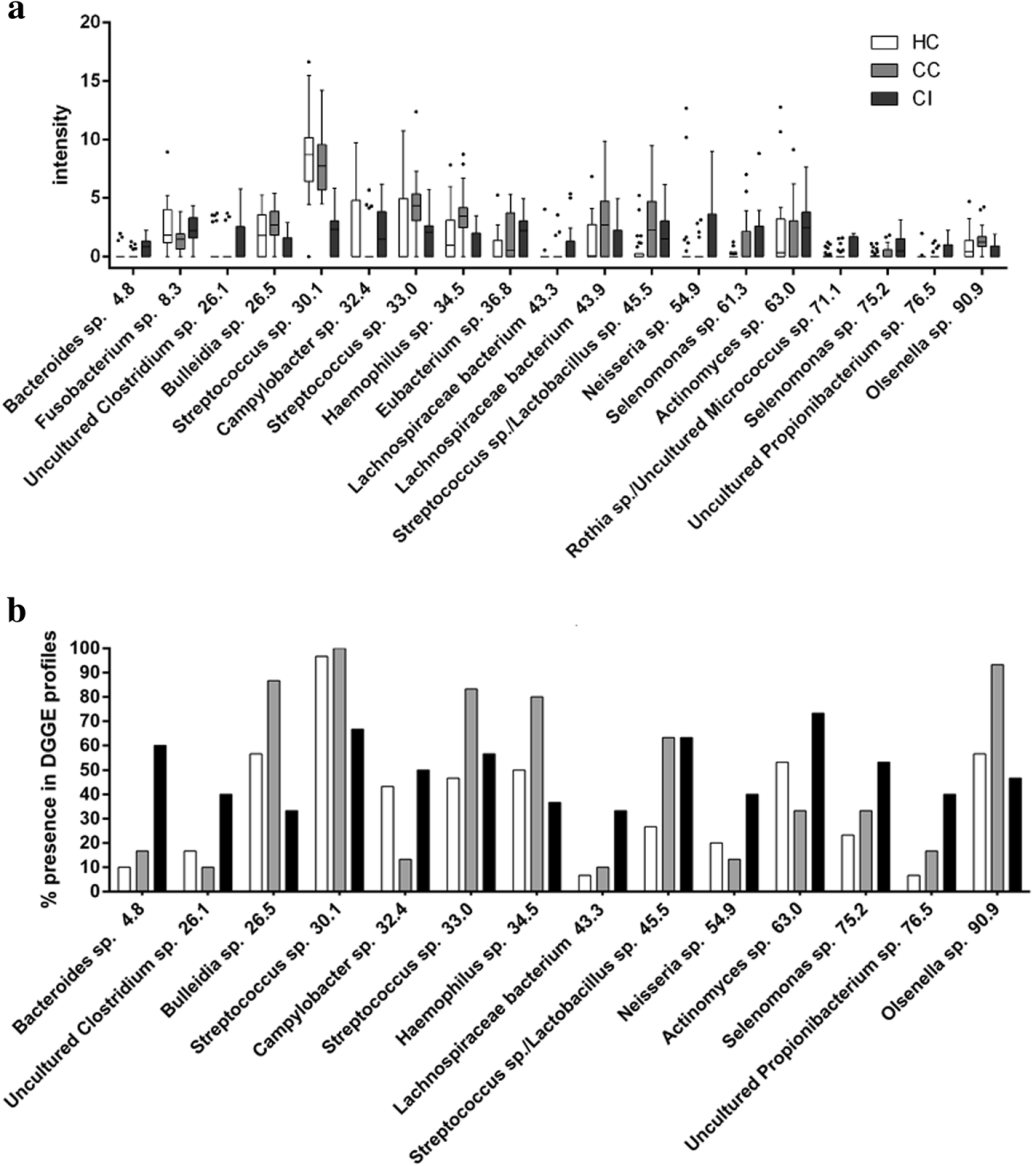
Identification and analysis of the key band-classes based on bacterial PCR-DGGE profiles of the groups. (**a**) Comparisons intensities of the key band-classes based on bacterial PCR-DGGE profiles of the different groups. (**b**) Analysis of the frequencies of key band-classes based on bacterial PCR-DGGE profiles of the different groups. (*P*-values for the comparison between the groups were shown in supplementary table S3)

To identify the key bacterial species that shifted composition, we calculated the intensity and the frequency of each band in the 90 PCR-DGGE profiles and analyzed the variation of each band-class. We found that the intensities of 19 band-classes differed significantly (Fig. 5a), and the frequencies of 14 band-classes varied (Fig. 5b) among the three groups. According to our analysis of the intensities and frequencies of the variable band-classes, six key band-classes were identified that reflected the differences between the CI and each control group. The abundances of five band-classes 4.8 (*Bacteroides* sp*.*), 36.8 (*Eubacterium* sp.), 43.3 (*Lachnospiraceae* sp*.*), 54.9 (*Neisseria* sp*.*), and 63.0 (*Actinomyces* sp*.*) were much higher in the group CI compared closely with those in each control group, while the abundance of band-class 30.1 (*Streptococcus* sp.) in group CI was significantly lower compared with each control group (*P* < 0.017 with modified Bonferroni correction the *P* –values were shown in Supplementary Table S3.)

To assess the effects of liver cirrhosis on band-class distribution, we analyzed the differences in the abundances of the band-classes between groups CC and HC and found that five band-classes, including 26.5 (*Bulleidia* sp*.*), 33.0 (*Streptococcus* sp.), 34.5 (*Haemophilus* sp*.*), 45.4 (*Lactobacillus* sp.), and 90.9 (*Olsenella* sp.) were higher in abundance in group CC versus group HC, while the abundances of band-classes 32.4 (*Campylobacter* sp.) and 63.0 (*Actinomyces* sp.) decreased in group CC versus group HC.

### Quantitative analysis of the disease-associated differences in the original (non-WGA) DNA

**Fig. 6:**
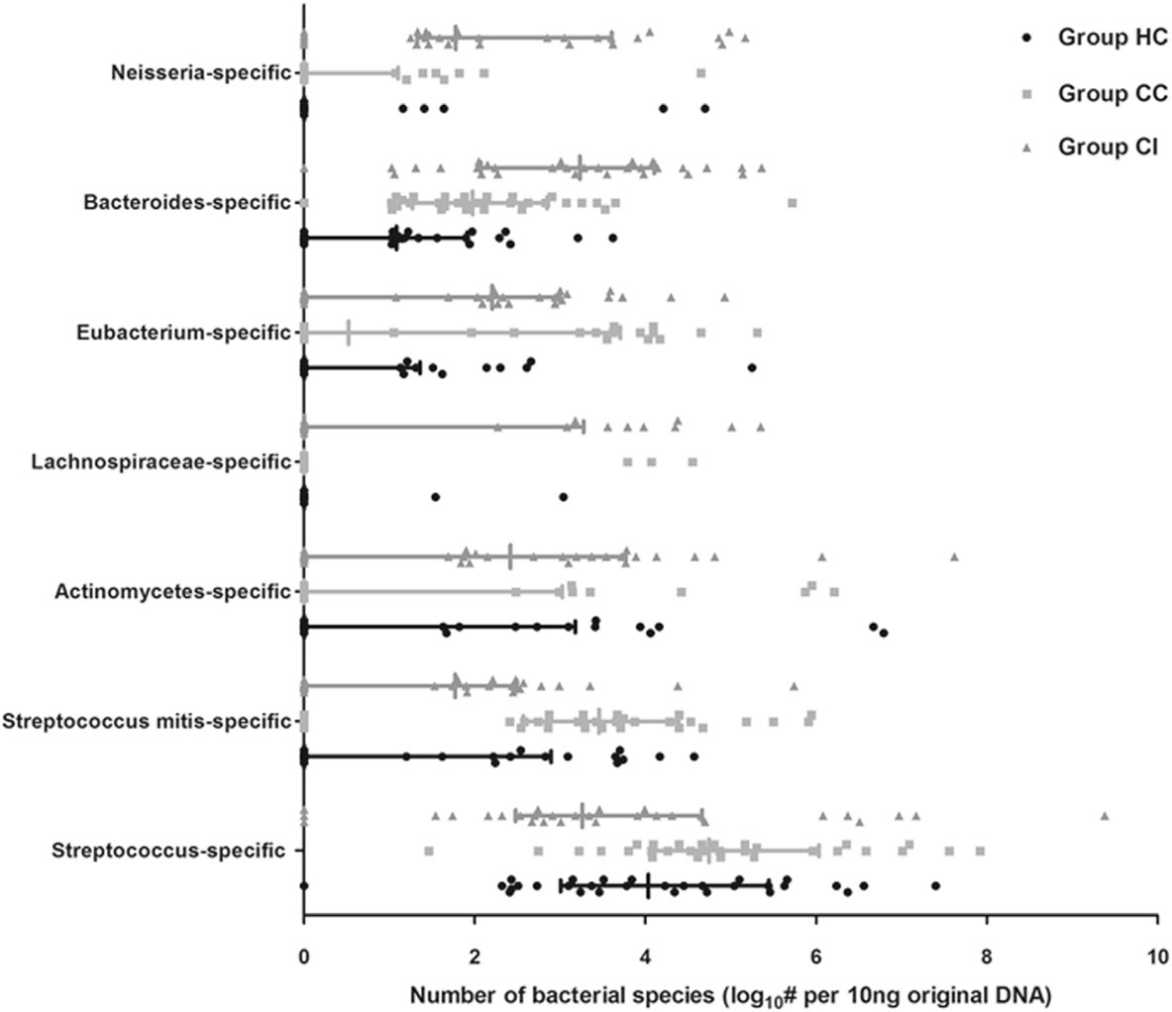
RT-qPCR analysis of the key bacterial species accounting for variations in microbiome compositions. Number of key bacteria: log # per 10 ng original swab DNA. The figure shows the numbers of key bacteria, including *Streptococcus*, *Streptococcus mitis*, *Actinomycetes*, *Lachnospiraceae*, *Eubacterium*, *Bacteroides*, and *Neisseria*

To verify the key bacteria present in infected subjects, which were associated with oropharyngeal microbial variation, quantitative PCR was used to analyze the original (non-WGA) DNA samples using bacterial species-specific primers. The abundances of *Bacteroides* sp., *Neisseria* sp., and *Actinomycetes* sp*.* and those of *Streptococcus* sp. in the oropharyngeal mucosa of group CI were higher and lower, respectively, compared with those of groups CC and HC (3.23, IQR 2.07–4.10 versus 1.08, IQR 0–1.91 and 1.97, IQR 1.27–2.85 for *Bacteroides* sp.; 1.78, IQR 1.33–3.60 versus 0, IQR 0–1.10 and 0 IQR 0–0 for *Neisseria* sp.; 2.42, IQR 0–3.77 versus 0, IQR 0–3.02 and 0, IQR 0–3.18 for *Actinomycetes* sp*.*; 3.26, IQR 2.48–4.66 versus 4.74, IQR 4.08–6.03 and 4.03, IQR 3.01–5.44 for *Streptococcus* sp*.*) (*p* < 0.05) (Fig. 6). The abundances of *Bacteroides* sp. and *Streptococcus mitis* were higher in group CC versus group HC (*p* < 0.05).

## Discussion

WGA enables discovery of genomic information in the field of microbial ecology that was not previously accessible [25]. In the present study, each swab of microbial DNA was amplified in triplicate using phi29 V2 DNA polymerase and analyzed using PCR-DGGE. The sequences of 39 band-classes representing commensal bacterial species were found to differ and were identified using BLAST to represent *Streptococcus*, *Actinomyces*, *Neisseria*, *Prevotella*, *Rothia*, *Veillonella*, *Eubacteriaceae*, and *Fusobacteria*. The oropharyngeal mucosal microbiome comprised *Firmicutes*, *Actinobacteria*, *Bacteroidetes*, *Proteobacteria*, and *Fusobacteria*, which is consistent with the results of others using high-throughput sequencing of the oral microbiome [26, 27].

The species listed above are always present at high frequency, typically in a balanced population in healthy individuals, and changes in their composition cause subsequent respiratory tract infections [28]. Therefore, the validity and reproducibility of the WGA method suggests that it is an appropriate technique for conducting subsequent PCR-DGGE analysis to determine and monitor the predominant differences in oropharyngeal microbiota in a large cohort of patients with respiratory tract infections.

The human microbiome, including oropharyngeal bacteria, is an essential component of immunity that influences pathogenesis [29] and determines the body’s physiological responses and susceptibility to disease [30]. Farrell and Zhang *et al.* [7] found that salivary microbiota serve as an informative source for discovering noninvasive biomarkers of pancreatic cancer and chronic pancreatitis. Here, our DGGE profiling results reveal considerable differences in the composition of oropharyngeal mucosal bacterial groups CI, CC, and HC. Further, increased diversity was detected in groups CI and CC compared with group HC, particularly for group CI. It is important to note that quantitative PCR analysis verified that the abundances of *Bacteroides* sp., *Neisseria* sp., and *Actinomycetes* sp*.* were high compared with that of *Streptococcus* sp. in group CI versus groups CC and HC. Therefore, we conclude that alterations of the populations of these four bacterial species in the oropharyngeal microbiome were associated with liver cirrhosis and pneumonia.

The mechanisms responsible for alterations of the microbiome are multifactorial and complex. First, viral infection modifies the systemic effects of the host by modulating the host’s immune response, which impairs mucosal immunity. Moreover, impaired local immunity and physical damage to the epithelium might enhance bacterial adherence and invasion. Accumulated evidence shows that infection is associated with polymicrobial interactions on mucosal surfaces that include commensal bacteria and exogenous pathogens [31]. For example, some pathogenic *Candida* species aggregate with *Fusobacterium* sp*.* [32], *Actinomyces* sp. [33], which may increase the colonization of mucosal epithelial cells.

Here, our quantitative PCR analysis indicates that the populations of *Bacteroides* sp., *Neisseria* sp., and *Actinomycetes* sp. increased dramatically and became the most prevalent species in group CI. These species originate in the oropharyngeal flora, and pathogenic and nonpathogenic *Bacteroides* sp. [34], *Neisseria* sp. [35] and *Actinomycetes* sp. [36] secrete outer-membrane vesicles that interact with neighboring cells through fusion or adherence. These microvesicles may impart a growth advantage, causing an imbalance in the composition of the oropharyngeal microbiota [37, 38] and subsequently impairing the barrier function of the mucosal biofilm, leading to the emergence of potentially pathogenic bacteria.

Moreover, host innate immune responses involve in competitive interactions between species and influence the structure and function of the flora [39]. Immune dysfunction is the most common clinical characteristic of patients with cirrhosis [40, 41] and is accompanied by alterations in the intestinal microbiota [42]. Intestinal microbes might interact with the microbiomes of other distant sites, including the respiratory tract and oral cavity [43] by activating the innate immune response. Therefore, we hypothesize that immune dysfunction might promote the growth of disease-associated oropharyngeal mucosal bacteria and subsequently cause an imbalance of oropharyngeal microbiota and enhanced susceptibility to infection by pathogens and facultative bacteria.

Moreover, the infection might inversely enhance the destruction of oropharyngeal microbiota. The destruction of oropharyngeal microbiota and infection might exert a synergetic effect on disease progression [31]. Strikingly, compared with group HC, the abundance of bacteria closely related to *Streptococcus* increased remarkably in group CC but decreased in group CI. In contrast, the population of bacteria closely related to *Actinomyces* decreased dramatically in group CC but increased in group CI. Bacterial colonization is determined by the ability to compete with co-inhabitants of a niche [44]. Understanding the importance of interspecies interactions and intra-species genetic and phenotypic variation might serve to control disease progression and influence treatment [45].

## Conclusions

The combination of WGA and DGGE analysis successfully monitored oropharyngeal microbial variations, and established that oropharyngeal microbiome of each subject maintained a relatively stable composition during the follow-up. However, it will be challenging to demonstrate a direct link between the species that populate the microbiota and the pathogenesis of pneumonia. Further studies using metagenomic approaches are required to identify the variations in more depth.

## Additional files

Additional file 1: Table S1. Questionnaire: Follow-up data for subjects who participated in the investigation of the oropharyngeal microbiome. Additional file 2: Table S2. The primers for qPCR in the study. Additional file 3: Table S3. Median intensity of band classless that differ significantly among HC CC and CI groups.

## Abbreviations

WGA: Whole genome amplification
DGGE: Denaturing gradient gel electrophoresis
qPCR: Quantitative polymerase chain reaction
Group CI: HBV decompensated cirrhotic patients with confirmed pneumonia
Group CC: HBV decompensated cirrhosis without any respiratory infection
Group HC: Healthy adult controls
UPGMA: The unweighted pair-group method with an arithmetic means
MDS: Multidimensional scaling
PCA: principal component analysis
