# Supplementary material for: Alterations of Bacteroides sp., Neisseria sp., Actinomyces sp., and Streptococcus sp. populations in the oropharyngeal microbiome are associated with liver cirrhosis and pneumonia
Source: BMC Infect Dis. 2015 Jun 23;15:239. doi: 10.1186/s12879-015-0977-x (PMC4477430; doi:10.1186/s12879-015-0977-x)
Supplement: Additional file 1: Table S1. — Questionnaire: Follow-up data for subjects who participated in the investigation of the oropharyngeal microbiome. [file 12879_2015_977_MOESM1_ESM.doc]

**Table S1.** Questionnaire

**Follow-up data for subjects who participated in the investigation of the oropharyngeal microbiome**

**Name： sex：**F/M **Date of birth (month-year)：**

**Telephone number(s)： Sample No:**

| Data of follow-up (Y-M-D) | | |  |  | | |  |
| --- | --- | --- | --- | --- | --- | --- | --- |
| Way of follow- up | | | 1. outpatient 2. inpatient | 1. outpatient 2. inpatient | | | 1. outpatient 2. inpatient |
| A survey  of disease symptoms | 1. Liver, lung, or previous related surgery  2. Basic diseases such as diabetes, coronary disease, hypertension  3. Ascites  4. Gastrointestinal bleeding  5. Liver cirrhosis  6. Infection  7. Respiratory symptoms: shortness of breath, sputum production, wheezing or chest pain or discomfort  8. Others | |  |  |  | | |
| X-ray examination | | | Examination day： | Examination day： | Examination day： | | |
|  |  |  |  |  |  |  |  |
| Oral health screening | | | Examination day： | Examination day： | Examination day： | | |
|  |  |  | 1.[periodontitis](http://dict.youdao.com/w/periodontitis/) □  2. oral ulcer □  3. cavities □  4. others: ______ | 1. [periodontitis](http://dict.youdao.com/w/periodontitis/) □  2. oral ulcer □  3. cavities □  4. others: ______ | 1. [periodontitis](http://dict.youdao.com/w/periodontitis/) □  2. oral ulcer □  3. cavities □  4. others: ______ | | |
| Biochemical blood routine inspection | | | Examination day： | Examination day： | Examination day： | | |
|  |  |  |  |  |  |  |  |
| Physical sign | Blood pressure (mmHg) | |  |  | | |  |
|  | Weight (kg) | |  |  | | |  |
|  | [Height](javascript:void(0);) (cm) | |  |  | | |  |
|  | Body mass index (BMI) | |  |  | | |  |
|  | Others | |  |  | | |  |
| Lifestyle | Daily consumption of cigarettes | |  |  | | |  |
|  | Daily consumption of alcohol | |  |  | | |  |
|  | Fitness habits | | Form:  Time: | Form:  Time: | | | Form:  Time: |
|  | DietaryHabits | Yoghurt |  |  | | |  |
|  |  | Staple |  | / | | | / |
|  | Psychological quality | | 1.better 2.good 3.worse | 1.better 2.good 3.worse | | | 1.better 2.good 3.worse |
|  | Treatment compliance | | 1.better 2.good 3.worse | 1.better 2.good 3.worse | | | 1.better 2.good 3.worse |
| Drugs | Antibiotics | |  |  | | |  |
|  | Probiotics | |  |  | | |  |
|  | Or others | |  |  | | |  |
|  | Drugs 1: (name) | |  |  | | |  |
|  | Drug dosage | | Times/Day:  Dosage/time: mg | Times/Day:  Dosage/time: mg | | Times/Day:  Dosage/time: mg | |
|  | Drugs 2:(name) | |  |  | |  | |
|  | Drug dosage | | Times/Day:  Dosage/time: mg | Times/Day:  Dosage/time: mg | | Times/Day:  Dosage/time: mg | |
|  | Drugs 3:(name) | |  |  | |  | |
|  | Drug dosage | | Times/Day:  Dosage/time: mg | Times/Day:  Dosage/time: mg | | Times/Day:  Dosage/time: mg | |
|  | Others | |  |  | | |  |
| Other need to supplement | | |  |  | | |  |
| Patient’s signature | | |  |  | | |  |
| Doctor’s signature | | |  |  | | |  |
| Date of next follow-up (year-month-day) | | |  |  | | |  |
