# Supplementary material for: Alterations of Bacteroides sp., Neisseria sp., Actinomyces sp., and Streptococcus sp. populations in the oropharyngeal microbiome are associated with liver cirrhosis and pneumonia
Source: BMC Infect Dis. 2015 Jun 23;15:239. doi: 10.1186/s12879-015-0977-x (PMC4477430; doi:10.1186/s12879-015-0977-x)
Supplement: Additional file 2: Table S2. — The primers for qPCR in the study. [file 12879_2015_977_MOESM2_ESM.doc]

**Table S2 Primers and conditions for qPCR used in the study**

| Target | Sequence (5′–3′) | Anneal (°C) | Ref. |
| --- | --- | --- | --- |
| *Lachnospiraceae*-specific | TTC GCA AGA ATG AAA CTC AAA G | 60 | [1] |
|  | AAG GAA AGA TCC GGT TAA GGA TC |  |  |
|  | (FAM) ACC AAG TCT TGA CAT CCG (minor groove binder [MGB]) |  |  |
| *Streptococcus*-specific | AGA GTT TGA TCC TGG CTC AG | 55 | [2] |
|  | GTA CCG TCA CAG TAT GAA CTT TCC |  |  |
| *Eubacterium*-specific | AGA GTT TGA TCC TGG CTC AG | 54 | [3] |
|  | GCC TTA AAC CCT RCG CTT |  |  |
| *Streptococcus mitis* | GCC GTC TCT TCT CGT TCT | 62 | [4] |
|  | GGA TTT TCA AGA TCA GCT ACC ATT |  |  |
| *Neisseria*-specific | CTG GCG CGG TAT GGT CGG TT | 60 | [5] |
|  | GCC GAC GTT GGA AGT GGT AAA G |  |  |
| *Actinomycetes*-specific | CCA GCA GCC GCG GTA AT | 55 | [6] |
|  | TCT GCG CAT TTC ACC GCT AC |  |  |
| *Bacteroides*-specific | TGG TAG TCC ACA CAG TAA ACG ATG A | 60 | [7] |
|  | CGT ACT CCC CAG GTG GAA TAC TT |  |  |
|  | (FAM) GTT TGC GAT ATA CAG TAA GCG GCC AAG CG |  |  |

**References**

1. Nowotny A, Behling UH, Hammond B, Lai CH, Listgarten M, et al.Release of toxic microvesicles by Actinobacillus actinomycetemcomitans. Infect Immun 1982; 37: 151-154.

2. Schertzer JW, Whiteley M. Bacterial outer membrane vesicles in trafficking, communication and the host-pathogen interaction. J Mol Microbiol Biotechnol 2013; 23: 118-130.

3. Kuehn MJ, Kesty NC. Bacterial outer membrane vesicles and the host-pathogen interaction. Genes Dev 2005; 19: 2645-2655.

4. Lysenko ES, Ratner AJ, Nelson AL, Weiser JN.The role of innate immune responses in the outcome of interspecies competition for colonization of mucosal surfaces. PLoS Pathog 2005; 1: e1.

5. Lansac N, Picard FJ, Menard C, Boissinot M, Ouellette M, et al. Novel genus-specific PCR-based assays for rapid identification of Neisseria species and Neisseria meningitidis. Eur J Clin Microbiol Infect Dis 2000; 19: 443-451.

6. Singh AK, Singh M, Dubey SK. Changes in Actinomycetes community structure under the influence of Bt transgenic brinjal crop in a tropical agroecosystem. BMC Microbiol 2013; 13: 122.

7. Brunk CF, Li J, Avaniss-Aghajani E. Analysis of specific bacteria from environmental samples using a quantitative polymerase chain reaction. Curr Issues Mol Biol 2002; 4: 13-18.
