# Supplementary material for: Alterations of Bacteroides sp., Neisseria sp., Actinomyces sp., and Streptococcus sp. populations in the oropharyngeal microbiome are associated with liver cirrhosis and pneumonia
Source: BMC Infect Dis. 2015 Jun 23;15:239. doi: 10.1186/s12879-015-0977-x (PMC4477430; doi:10.1186/s12879-015-0977-x)
Supplement: Additional file 3: Table S3. — Median intensity of band classless that differ significantly among HC CC and CI groups. [file 12879_2015_977_MOESM3_ESM.docx]

| **Table S3**. Median intensity of band classes that differ significantly among HC CC and CI groups | | | | | | | | | | | |
| --- | --- | --- | --- | --- | --- | --- | --- | --- | --- | --- | --- |
| Band | Band class | Closest BLAST match | Median intensity (IQR) | | | p value | | | Result | | |
|  |  |  | HC n=30 | CC n=30 | CI n=30 | HC vs.CC | CC vs.CI | HC vs.CI | HC vs.CC | CC vs.CI | HC vs.CI |
| 14 | 4.80% | Bacteroides ovatus | 0（0-0） | 0（0-0） | 0.80（0-1.29） | 0.573 | 0 | 0 |  | CC<CI | HC<CI |
| 1 | 8.30% | Fusobacterium sp. | 1.82（1.15-3.98） | 1.46（0.62-1.90） | 2.18（1.58-3.30） | 0.031 | 0.003 | 0.923 | HC>CC | CC<CI |  |
| 24 | 26.10% | Uncultured Clostridium sp. | 0（0-0） | 0（0-0） | 0（0-2.53） | 0.476 | 0.013 | 0.077 |  | CC<CI |  |
| 29 | 26.50% | Bulleidia sp. | 1.79（1-3.54） | 2.68（1.81-3.84） | 0（0-1.58） | 0.095 | 0 | 0.008 |  | CC>CI | HC>CI |
| 6 | 30.10% | Streptococcus sp. | 8.70（6.42-10.17） | 7.75（5.70-9.57） | 2.30（0-3.01） | 0.301 | 0 | 0 |  | CC>CI | HC>CI |
| 7 | 32.40% | Campylobacter sp. | 0（0-4.77） | 0（0-0） | 1.46（0-3.82） | 0.011 | 0.006 | 0.968 | HC>CC | CC<CI |  |
| 19 | 33.00% | Streptococcus mitis | 0（0-4.93） | 4.34（3.04-5.33） | 2.04（0-2.61） | 0.039 | 0 | 0.594 | HC<CC | CC>CI |  |
| 25 | 34.50% | Haemophilus sp. | 0.95（0-3.08） | 3.45（2.46-4.15） | 0（0-1.97） | 0.008 | 0 | 0.073 | HC<CC | CC>CI |  |
| 15 | 36.80% | Eubacterium sp. | 0（0-1.36） | 0.52（0-3.71） | 2.20（0-3.00） | 0.118 | 0.837 | 0.003 |  |  | HC<CI |
| 18 | 43.30% | Lachnospiraceae bacterium | 0（0-0） | 0（0-0） | 0（0-1.28） | 0.644 | 0.037 | 0.01 |  | CC<CI | HC<CI |
| 33 | 43.90% | Lachnospiraceae bacterium | 0.04（0-2.70） | 2.66（0-4.72） | 0（0-2.22） | 0.077 | 0.01 | 0.347 |  | CC>CI |  |
| 30 | 45.50% | Lactobacillus casei/Streptococcus sobrinus | 0（0-0.24） | 2.24（0-4.68） | 1.48（0-3.01） | 0.001 | 0.392 | 0.002 | HC<CC |  | HC<CI |
| 26 | 54.90% | Neisseria sp. | 0（0-0） | 0（0-0） | 0（0-3.60） | 0.546 | 0.01 | 0.063 |  | CC<CI |  |
| 21 | 61.30% | Selenomonas sp. | 0（0-0） | 0（0-2.13） | 0（0-2.58） | 0.006 | 0.685 | 0.052 | HC<CC |  |  |
| 5 | 63.00% | Actinomyces sp. | 0.32（0-3.18） | 0（0-3.03） | 2.42（0-3.77） | 0.292 | 0.017 | 0.068 |  | CC<CI |  |
| 34,28 | 71.10% | Rothia sp. /Uncultured Micrococcus sp. | 0（0-0.04） | 0（0-0） | 0（0-1.67） | 0.886 | 0.013 | 0.018 |  | CC<CI | HC<CI |
| 10 | 75.20% | Selenomonal sp. | 0（0-0.06） | 0（0-0.56） | 0.44（0-1.50） | 0.343 | 0.024 | 0.002 |  | CC<CI | HC<CI |
| 22 | 76.50% | Uncultured Propionibacterium sp. | 0（0-0） | 0（0-0） | 0（0-0.98） | 0.233 | 0.03 | 0.002 |  | CC<CI | HC<CI |
| 35 | 90.90% | Olsenella sp. | 0.40（0-1.37） | 1.22（0.84-1.68） | 0（0-0.87） | 0.003 | 0 | 0.297 | HC<CC | CC>CI |  |
| P < 0.017 with modified Bonferroni correction | | | | | | | | | | | |
